# Epidemiology and Clinical Features of Patients with Visceral Leishmaniasis Treated by an MSF Clinic in Bakool Region, Somalia, 2004–2006

**DOI:** 10.1371/journal.pntd.0000085

**Published:** 2007-10-31

**Authors:** Marie-Eve Raguenaud, Anna Jansson, Veerle Vanlerberghe, Geert Van der Auwera, Stijn Deborggraeve, Jean-Claude Dujardin, Giannos Orfanos, Tony Reid, Marleen Boelaert

**Affiliations:** 1 Médecins Sans Frontières, Medical Department, Brussels, Belgium; 2 Médecins Sans Frontières, Somalia Mission, Nairobi, Kenya; 3 Epidemiology and Disease Control Unit, Institute of Tropical Medicine, Antwerp, Belgium; 4 Parasitology Department, Institute of Tropical Medicine, Antwerp, Belgium; Sabin Vaccine Institute, United States of America

## Abstract

**Background:**

There are few reports describing the epidemiology of visceral leishmaniasis (VL) in Somalia. Over the years 2002 to 2005, a yearly average of 140 patients were reported from the Huddur centre in Bakool region, whereas in 2006, this number rose to 1002 patients. Given the limited amount of information on VL and the opportunity to compare features with the studies done in 2000 in this part of Somalia, we describe the epidemiologic and clinical features of patients who presented to the Huddur treatment centre of Bakool region, Somalia, using data routinely collected over a five-year observation period (2002–2006).

**Methodology:**

Methods used included the analysis of routine data on VL cases treated in the Huddur treatment centre, a retrospective study of records of patients admitted between 2004 and 2006, community leaders interviews, and analysis of blood specimens taken for parasite species identification in Antwerp Institute of Tropical Medicine.

**Principal Findings:**

A total of 1671 VL patients were admitted to the Huddur centre from January 2002 until December 2006. Nearly all patients presented spontaneously to the health centre. Since 2002, the average patient load was stable, with an average of 140 admissions per year. By the end of 2005, the number of admissions dramatically increased to reach a 7-fold increase in 2006. The genotype of *L. donovani* identified in 2006 was similar to the one reported in 2002. 82% of total patients treated for VL originated from two districts of Bakool region, Huddur and Tijelow districts. Clinical recovery rate was 93.2% and case fatality rate 3.9%.

**Conclusions:**

After four years of low but constant VL case findings, a major increase in VL was observed over a 16-month period in the Huddur VL centre. The profile of the patients was pediatric and mortality relatively low. Decentralized treatment centers, targeted active screening, and community sensitization will help decrease morbidity and mortality from VL in this endemic area. The true magnitude of VL in Somalia remains unknown. Further documentation to better understand transmission dynamics and thus define appropriate control measures will depend on the stability of the context and safe access to the Somali population.

## Author Summary

Our paper describes the epidemiological features of visceral leishmaniasis in the Bakool region, South Central Somalia, over the years 2004 to 2006. Since 2000, Médecins Sans Frontières has been providing care for patients suffering from visceral leishmaniasis in Huddur, located in a region endemic for visceral leishmaniasis. By the end of 2005, we witnessed a dramatic increase in the number of patients admitted to the Huddur centre with visceral leishmaniasis. In our paper, we provide a description of the profile of patients admitted, thus giving an insight into the epidemiology of visceral leishmaniasis in a part of the world where relatively little has been documented and where the true magnitude of this neglected disease remains unknown.

## Introduction

Visceral leishmaniasis (VL) is a vector-borne parasitic disease caused by *Leishmania donovani.* According to WHO, over the last 15 years, endemic regions have been extending and there has been a sharp increase in the number of recorded cases of the disease. For example, in eastern African countries it has caused epidemic outbreaks like the ones that occurred in Southern Sudan from 1984–1994 [1], in North-eastern Kenya and South-eastern Ethiopia in 2000–1, in eastern Sudan from 1996–97 [2, in Ethiopia and Eritrea in 1997–98 3]. Much of VL is concentrated in East Africa [4] yet little has been reported from the endemic parts of Somalia.

Different profiles of patients with VL and outcomes have been described in Africa. In Ethiopia VL is commonly observed as an opportunistic infection in HIV infected adults with documented mortality rates up to18.5% [5]. In Western Upper Nile, Sudan, the majority of cases reported during a major outbreak from 1984 to 1994 were adults with death rates of 38–57% [1]. In other regions of Sudan and in West Pokot of Uganda it presents mainly as a pediatric problem [6]. In the endemic area of Baringo district in Kenya changing lifestyle has led to a decreasing proportion of new VL cases among men [7].

Areas of Somalia where VL has been reported include the coastal areas in the south of the country [8,9], the area along the Shebelle river in the south of Somalia 10], Lower Juba region (MSF, unpublished report), and Baidoa in Bay region [11]. Information on local vector behaviour and risk factors for infection or disease in Somalia are very limited. In Somalia transmission is thought to be anthroponotic similar to other endemic areas of the region (Uganda, Southern Sudan, Kenya) [12,6]. A study in Kenya revealed that transmission occurs in and around houses [7], but whether this occurs in Somalia is unknown. Termite hills are the favoured breeding and resting sites of *P.martini* and they are very common in Bakool [13,14].

The turmoil and factional fighting that followed the regime's overthrow in 1991 has left large parts of Somalia without any form of health care. Even in 2006, the majority of health care provided in South Central Somalia is carried out by non-governmental organizations – but with very limited coverage of the Somali population.

Bakol region is located in south-central Somalia, bordering with Hiiraan region to the east, Bay region to the south, Gedo region to the west, and Ethiopia to the north. Médecins Sans Frontières (MSF), a private, non-governmental organization, has been working in Huddur, the capital of Bakool region since 2000, running a primary health care project consisting of outpatient and in-patient departments, a therapeutic feeding centre, a tuberculosis and a VL program. It has been the only treatment centre for VL in Bakool region until 2006. The first report published in the medical literature about VL in the Bakool region in 2000–01 concluded from the pediatric profile of the disease and information obtained from qualitative methods that VL was since long endemic in that region [15]. The infectious agent was confirmed as *L.donovani,* and entomological studies revealed the presence of potential vectors, *Phlebotomus martini* and *Phlebotomus vansomerenae* in Bakool region [16]. In the first year that VL was treated in Huddur - between July 2000 and August 2001- 230 patients with VL were identified and treated [15]. Since 2002 the average caseload was stable at around 140 VL cases per year until September 2005 when an increase in admissions was observed. A total of 1002 patients representing a seven-fold increase compared to previous years average were diagnosed in year 2006.

Given the limited amount of information on VL and the opportunity to compare features with the studies done in 2000 in this part of Somalia, we describe the epidemiologic and clinical features of patients who presented to the Huddur treatment centre of Bakool region, Somalia, using data routinely collected over a five-year observation period (2002–2006).

## Methods

We describe the profile of VL in the Bakool region using several methods: an analysis of epidemiological and clinical data from VL cases treated in a VL-treatment centre, using a retrospective analysis of case records, and analysis of blood specimens.

### Setting and population

The Bakool region consists of 5 districts: Huddur, Tijeglow, Rabdhure, Wajid and El-Berde, with a total estimated population of 245 000. Most of the population of Bakool region have a semi-nomadic lifestyle. The health centre in Huddur run by MSF serves as the primary provider of medical care for the population living in Huddur district. The population described in this report included all patients diagnosed with VL at the Huddur health centre between January 2002 and December 2006. All patients with VL were treated as in-patients in the VL ward of the centre for the entire duration of treatment of one month.

### Definitions and data collection

#### A: Diagnosis, treatment, and discharge

Standardized definitions for clinical and confirmed VL were used by the clinical officers as per written clinical protocol (MSFB Field Guidelines for VL, 2004). The clinical case definition of VL was: any patient reporting a history of fever for more than 2 weeks in combination with splenomegaly (palpable spleen), elevated temperature (>38.5°C) or wasting on clinical exam, in whom malaria had been excluded (negative Paracheck test or malaria treatment given).

In all patients meeting the clinical case definition, VL was confirmed by a positive serological test, either direct agglutination test (DAT, titre>1∶800) or rK39 dipstick test (Optileish®, Diamed, Switzerland) [17,18]. Serological diagnostic tests DAT and Optileish were used, which have a sensitivity of 96.9% and 90.1% and a specificity of 93.7% and 93.1% respectively [19]. DAT was performed by the AMREF laboratories based in Nairobi, Kenya from blood samples taken on filter paper. In September 2004 a new diagnostic test, the rK39 dipstick, was available and introduced in Huddur centre. It permitted confirmation of VL in the field. Critically ill patients who had features consistent with the clinical case definition but with negative DAT and Optileish dipstick were put on treatment based at the doctor's discretion. Spleen aspiration was not performed because it was considered too risky in this health setting. The treatment for primary VL as per WHO guideline [20] was sodium stibogluconate (SSG, produced by Albert David Ltd, Calcutta, India) as a daily intramuscular injection at a dosage of 20 mg/kg bodyweight for 30 days. Clinical features on admission and during treatment were recorded for each patient in a standardized patient file, from which the clinical information was encoded in an Access-based software developed by Epicentre (42 bis Boulevard Richard Lenoir, 75011 Paris).

Standardized definitions used for treatment outcome were included in the clinical protocol: clinical recovery at the end of treatment was defined as ‘no unexplained fever in the last two weeks of the treatment, regression of the spleen, no bleeding, weight gain and improved general well being’ (MSFB Field Guidelines for VL, 2004).

#### B: Confirmation laboratory study

A convenience sample of blood samples for species identification was taken from 17 Optileish positive patients admitted to the Huddur centre in August 2006. This was done in order to compare the genotype of the parasites detected in 2006, with the ones identified from patient samples taken in year 2000 by MSF.

Samples were mixed with the same volume of AS1-buffer (Qiagen) and sent to the Institute of Tropical Medicine, Antwerp, Belgium. DNA extraction was performed with the QIAamp DNA blood Mini Kit according to manufacturer's instructions (Qiagen). The samples were analyzed for the presence of *Leishmania* DNA by using a *Leishmania* specific polymerase chain reaction (PCR) that amplifies a 120 bp fragment of the parasite 18S ribosomal RNA gene (Deborggraeve *et al*., unpublished results); this gene target has been reported elsewhere for the sensitive and specific diagnostics of *Leishmania* infections [21[. Species identification was done by two species-specific PCR assays [22], L.infantum- and L.donovani- specific, respectively targeting the E and F copies of the cysteine proteinase B gene (cpb). These copies are situated at the 3′ end of the cpb gene cluster, and the type of gene is indicative for the species: *L. infantum* strains are characterized by cpbE, while cpbF is found only in *L. donovani* parasites [23]. Relative to cpbF, cpbE contains a deletion of 39 nucleotides (13 amino acids), a feature used for the design of the specific primers. The reference strains MHOM/MA/67/ITMAP263 (*L. infantum*) and MHOM/IN/2000/DEVI (*L. donovani*) were used as controls. Species identification was confirmed by PCR-restriction fragment length polymorphism (PCR-RFLP) [24].

### Data entry and analysis

Since the VL treatment centre was established, data from patients was collected by one national clinical officer who has been working continuously on the program. The total number of admissions was obtained from the main registration book of the treatment centre. Demographic and clinical information were collected from individual patient cards. Data from these patient cards was entered routinely in the Access-based Kala Azar Software that was first introduced in the MSF centre the field in year 2004. Clinical records of patients admitted before 2004 were not available for retrospective data entry. A total of 970 patient records were entered into the software from January 2004 until December 2006. This data was then exported to Excel program for data management and analyses. We analyzed the number of cases of VL detected over time, by age, sex, and geographical origin. We compared clinical features between patients admitted during the low case detection period before September 2005 (Jan04–Aug05) and the high case detection period starting in September 2005 when the first increase in admissions was noticed (Sep05–Dec06). Chi-squared test was used to compare proportions. Student t-test was used to compare means.

### Ethics

This outbreak investigation was viewed as a routine operational response. Ethical issues were addressed the following way: we used only routinely collected data in the process of monitoring a treatment program, confidentiality of clinical and laboratory patient information was maintained, patients were explained the reason for taking additional blood samples and were asked for oral consent, and blood samples analyzed in Antwerp Tropical Institute were only used only to detect the parasites and perform species identification. There are no laboratories in the southern Somalia doing parasitological tests. The Ethics Review Board instituted by Médecins Sans Frontières reviewed that relevant ethical issues in this project were well considered.

## Results

### Treatment centre admissions

A total of 1671 VL patients were admitted for treatment in the Huddur Health Centre from Jan 2002 until December 2006. Except for 27 found during outreach activities, all patients presented spontaneously to the health centre. See Table 1 for the number of admissions per year and the number of clinical records available. Clinical records which were entered in the Kala azar Software in 2004–2006 were available for a total of 970 patients. The incomplete data entry in 2005–6 is attributed to the loss of patient cards during the high workload period when record keeping became secondary to patient care.

**Table 1:** Number of VL patient admissions per year and number of clinical records available for analysis.

Year	Number of patients admitted	Number of patient records available (%)
2002	175	0
2003	107	5 (5%)
2004	117	117 (100%)
2005	270	166 (61%)
2006	1002	682 (68%)

**Figure 1:**
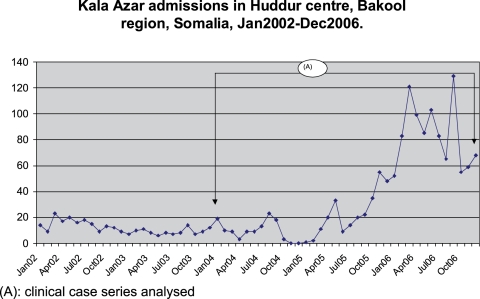
Number of VL cases diagnosed in MSF centre in Huddur from January 2002 to December 2006.

Figure 1 illustrates the number of VL patients admitted for treatment in Huddur centre across time since January 2002. After a period of relatively low caseload with usually fewer than 20 admissions per month (Jan02 until Oct04), there were 5 months of very low patient admissions due to the absence of the expatriate team (Nov04 until Mar05), during which patients were accepted on an exceptional basis. In September 2005 the monthly case detection started increasing to reach two peaks of over 100 admissions in March–April06 and in September06. Although the number of admissions was dropping after September 2006, the number of admissions in the last quarter of 2006 still remained higher than what was observed in previous years.

**Figure 2:**
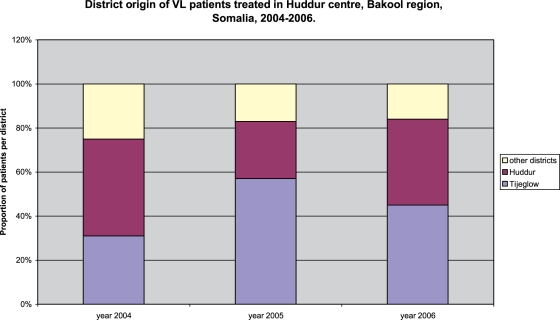
District of origin of VL patients treated in Huddur centre, Bakool region, Somalia, 2004–2006.

Information on place of origin was available for 905/970 patients. Although patients with VL originated from all 5 districts of Bakool region as well as from Baidoa district of Bay region (6%), the majority of patients originated from only 2 districts, Huddur and Tijeglow throughout the years as seen in Figure 2.

**Figure 3:**
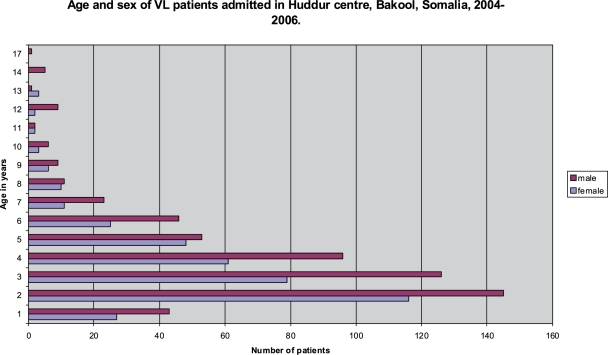
Age and sex of VL patients admitted in Huddur centre, Bakool, Somalia, 2004–2006.

Figure 3 shows the age and sex distribution for 969 patients. Median age of patients was 3.8 years (inter-quartile range 2 to 5) and overall boys represented 59.4% of all patients. No adults were diagnosed.

### Clinical features (Table 2)

**Table 2:** History and clinical features on admission for VL during the low (Jan04–Aug05) and high case detection periods (Sep05–Dec06), Huddur centre, Bakool, Somalia.

	“low caseload” Dec03–Aug05	“high caseload” Sep05–Dec06	P-value
	N = 146	N = 824	
Average duration of illness before admission	4.3 months (N = 131)	3.8 months (N = 810)	P = 0.09
History of fever	138/140 *(98.6%)*	811/820 *(98.9%)*	P = 0.49
History of epistaxis	21/74 *(28.4%)*	198/721 *(27.5%)*	P = 0.97
Loss of appetite	53/62 *(85.5%)*	551/749 *(73.6%)*	**P<0.001**
Cough	113/132 *(85.6%)*	681/790 *(86.2%)*	P = 0.85
Diarrhea	6/133 *(4.5%)*	69/806 *(8.6%)*	P = 0.11
Vomiting	20/133 *(15.0%)*	169/805 *(21.0%)*	P = 0.11
Average MUAC* (mm)	123.8 *(Range: 96–168)*	123.6 *(Range: 78–170)*	P = 0.87
MUAC <110 mm (severe malnutrition)	14/119 *(11.8%)*	86/630 *(13.6%)*	P = 0.58
MUAC <126 mm (moderate malnutrition)	50/119 *(42.0%)*	544/630 *(86.3%)*	**P<0.001**
Average Weight (Kg)	11.5 *(Range: 6.5–24.0)*	10.8 *(Range: 4.2–35.0)*	P = 0.032
Clinical fever (>37.5°C)	125/146 *(85.6%)*	501/818 *(61.2%)*	**P<0.001**
Average Temperature on admission (°C)	38.34	37.8	**P<0.001**
Clinical anemia	118/143 (*82.5%)*	624/789 *(79.1%)*	P = 0.35
Jaundice	2/125 (*1.6%)*	8/293 *(2.7%)*	P = 0.49
Edema	14/13 *(10.8%)*	101/468 *(21.6%)*	**P = 0.006**
Average Spleen size on admission (cm)	8.5 (N = 131)	6.4 (N = 717)	**P<0.001**
Lymph node enlargement	0/14 *(0%)*	12/184 *(6.5%)*	P = 0.40
Hepatomegaly	23/42 *(54.8%)*	86/378 *(22.7%)*	**P<0.001**

MUAC: Middle Upper Arm Circumference.

From the 970 clinical series entered in the database, we had laboratory results for 943 patients and out of these 916 (97.1%) were serologically confirmed with either DAT or the Optileish dipstick. Post Kala Azar dermal leishmaniasis was exceptional with only 1 case diagnosed in 2006. The overwhelming majority of patients admitted were new cases with only 6 clinical relapses diagnosed in 2006 and none in 2005. The core clinical features of VL were commonly observed: fever, splenomegaly, weight loss/wasting, and clinical anemia. Cough, epistaxis, and vomiting were frequently reported accompanying symptoms. Diarrhea, jaundice, and lymph node enlargement were infrequent. Duration of illness before first consultation was around 4 months. Variables which showed significant differences in frequencies between patients admitted during the low (Jan04–Aug05) and high case detection period (Sep05–Dec06) included loss of appetite, moderate malnutrition, clinical fever on admission, edema, and average spleen size on admission. Moderate malnutrition, as measured by a Middle Upper Arm Circumference (MUAC) less than 126 mm on admission, was much less prevalent among patients during the low case detection period compared to the high one (42.0% compared to 86.3%; P-value<0.001). The proportion of patients with clinical fever (>37.5°C) measured at admission was higher during the low case detection period than the high case detection period (85.6% compared to 61.2%; P-value<0.001). Average spleen size was higher during low case detection period compared to the high one (8.5 cm compared to 6.4 cm; P-value<0.001). Hepatomegaly was more frequent in low case detection period than the high one (54.8% compared to 22.7%; P-value<0.001) but data was only available for 42 patients during the low case detection period.

### Treatment and outcomes

Information on treatment outcome was available for 925 of the 970 case series studied (95.4%). A total of 36 deaths were recorded in the case series from January 2004 to December 2006, giving an overall case-fatality rate of 3.9%. Clinical recovery rate was 93.2% (862/925). A total of 27 patients defaulted (2.9%).

### Parasite characterization

DNA of *Leishmania* spp. was found in 12 of the 17 samples taken from Optileish positive patients under SSG treatment (range of 1 to 9 days of treatment). In 3 of them species identification was reached and confirmed the presence of *L.donovani*. The 12 samples were negative with the *L.infantum*-specific PCR. The 9 negative samples with the *L.donovani*-specific PCR were likely due to a lower sensitivity of the species-specific PCR in comparison with the diagnostic PCR. For 2 of the *L.donovani* positive samples, *cpb* PCR-RFLP patterns could be visualised and appeared to be similar to the ones encountered in the *L.donovani* samples taken in year 2000 [15]. PCR positive samples belonged to patients living in the districts of Huddur, Tijeglow, and Baidoa district of Bay region.

## Discussion

This report describes the pattern of VL admissions over a five-year period and the clinical characteristics and outcomes of 970 patients treated for VL in Huddur centre run by MSF in Somalia over a 3-year period. After four years of low but constant passive VL case finding in the endemic area of Bakool region, a major increase in VL patient admission was observed over a 16-month period in the Huddur centre. Although the reported number of patients treated gives an underestimate of the real prevalence, the trend in case detection clearly shows a sharp increase during the past 16 month period (Sep05–Dec06). The number of patient admissions was not found to be subject to seasonal variation. 82% of total patients treated for VL originated from two districts of Bakool region, Huddur and Tijeglow districts. This could reflect a real clustering of VL as is known to occur within endemic areas. For instance in Baringo district, Kenya, important differences in seroprevalences between villages of the same endemic area were documented [25]. There could be specific environmental and social factors in the group of villages most affected in Tijeglow district that favour transmission, but we have no information on it.

The pediatric profile of patients suggests that adults in the area are immune and that we are dealing with an endemic pattern of VL. The DNA pattern of *L.donovani* identified in 2006 was similar to the one identified in 2000 [15] suggesting that the same parasite strains remain circulating in the area over the period separating the two sample collections. We cannot exclude the existence of new parasite variants that would have been introduced in the area, but to explore this possibility, further and extensive molecular analysis would be required.

Better awareness amongst the population of the treatment availability, spread by successfully treated patients, may have contributed to the rise of detected cases. But the five-fold increase in the monthly case detection implies that other factors came into play and we cannot exclude a real increase in disease because the prevalence of VL in the area is unknown.

Clinical features observed were typical of VL. The possible increase in the population's knowledge on the disease and on the availability and reputation of treatment in Huddur, may have contributed to the reduced average duration of sickness before presentation: 4 months compared to 19 months in year 2000–1 [15]. This short duration of illness could explain the low case fatality rate (3.9%) as found in Sudan [12]. We cannot explain the observed sex difference among VL patients treated in Huddur centre but suggest the boys had greater exposure to sand fly bites of boys and/or possibly better access to care.

Although, statistically, there was no difference in duration of illness, this may be due to lack of power, since the clinical differences between patients of low and high caseload periods suggest a shorter duration of illness in patients admitted during the high caseload period. However, the difference in hepatomegaly is probably artificial: missing values most likely represent negative clinical findings, which if added to the denominator would reduce the proportion of hepatomegaly during the low caseload period. The fact that malnutrition was more common among patients admitted during the high case detection period compared to the low case detection period would suggest that a worsening in the nutritional status among children in Tijeglow and Huddur districts could have contributed to the increase in cases of of VL. Malnutrition is a well known risk factor in the development of VL disease [26].

Based on our experience certain measures could be implemented for improved care. Lack of transport and the long distances to travel are known to limit the physical access to Huddur centre. Opening other temporary treatment centers especially in the most affected districts could improve access to care for VL patients. Active screening for VL, when doing outreach work such as for vaccination or nutritional screening, would help further increase the number of cases detected and treated. Sadly, the instability in the area due to armed conflict seriously thwarts these efforts. Shorter treatment regimens are needed and would greatly help improve acceptability of treatment and increase the treatment completion rate.

Vector control and other preventive measures have not been implemented but could improve control of VL in the area. For instance the use of treated bed-nets to protect from the *P.martini* bites, active at night time, which would not only reduce in-household transmission of VL but also of malaria. Targeted information and education of the population to increase awareness could help increase early case detection and limit the use of traditional remedies like abdominal scarification. Insecticidal application to termite mounds could be a measure of targeted control in the most affected villages.

There are a number of significant limitations to our data. One was the lack of full clinical records. Although we cannot exclude bias, there is no reason to believe that the missing patient charts are associated with a particular patient characteristic or outcome as missing data was likely due to reduced documentation of patient charts due to high patient load. The constrained time and access to remote villages did not allow for a prevalence survey in the whole area affected by VL.

As exposure to sandfly varies from area to area, a case-control study to determine the local risk factors of VL would be useful to define targeted control measures. Additional documentation on rain patterns, vector behaviour, and other risk factors for VL like HIV co-infection would be useful in designing adapted interventions to decrease morbidity and mortality.

Our experience suggests that VL is substantially underreported in Bakool region and possibly in neighboring regions of southern Somalia. The true magnitude of the problem of VL in Somalia is likely to remain unknown and documentation and implementation of effective interventions to control VL will be limited as long as there will be no safe access to population and inexistent health care services.
