# Correction: Epidemiology and Clinical Features of Patients with Visceral Leishmaniasis Treated by an MSF Clinic in Bakool Region, Somalia, 2004–2006

**DOI:** 10.1371/journal.pntd.0000195

**Published:** 2008-02-06

**Authors:** Marie-Eve Raguenaud, Anna Jansson, Veerle Vanlerberghe, Gert Van der Auwera, Stijn Deborggraeve, Jean-Claude Dujardin, Giannos Orfanos, Tony Reid, Marleen Boelaert

## Original Article

Raguenaud M, Jansson A, Vanlerberghe V, Van der Auwera G, Deborggraeve S, et al. (2007) Epidemiology and Clinical Features of Patients with Visceral Leishmaniasis Treated by an MSF Clinic in Bakool Region, Somalia, 2004–2006. PLoS Negl Trop Dis 1(1): e85. doi:10.1371/journal.pntd.0000085

## Correction

An author name was misspelled: Geert Van der Auwera should be spelled Gert Van der Auwera.

