# Correction: Epidemiology and Clinical Features of Patients with Visceral Leishmaniasis Treated by an MSF Clinic in Bakool Region, Somalia, 2004–2006

**DOI:** 10.1371/journal.pntd.0009356

**Published:** 2021-04-19

**Authors:** Marie-Eve Raguenaud, Anna Jansson, Veerle Vanlerberghe, Gert Van der Auwera, Stijn Deborggraeve, Jean-Claude Dujardin, Ioannis Orfanos, Tony Reid, Marleen Boelaert

The seventh author’s name is spelled incorrectly. The correct name is: Ioannis Orfanos.
